# A Importância de se Entender a Evolução da Fibrose Miocárdica na Cardiomiopatia Chagásica Crônica

**DOI:** 10.36660/abc.20210897

**Published:** 2021-11-22

**Affiliations:** 1 Universidade de São Paulo Faculdade de Medicina Hospital das Clínicas São Paulo SP Brasil Universidade de São Paulo, Faculdade de Medicina, Hospital das Clínicas - Instituto do Coração, São Paulo, SP – Brasil; 2 Hospital do Coração São Paulo SP Brasil Hospital do Coração (HCOR), São Paulo, SP – Brasil

**Keywords:** Cardiomiopatia Chagásica, Fibrose Endomiocárdica, Doença de Chagas, Insuficiência Cardíaca, Diagnóstico por Imagem/métodos, Imagem por Ressonância Magnética/métodos

A fibrose miocárdica é um dos biomarcadores mais importantes da cardiomiopatia chagásica, e está diretamente relacionada à gravidade da cardiomiopatia e ao estágio da doença.^1^ Além disso, a fibrose miocárdica tem valor prognóstico para eventos cardiovasculares, com uma forte relação com eventos arrítmicos nesses pacientes.^2-4^

A ressonância magnética cardíaca (RMC) é o método de imagem de referência para detecção e quantificação de fibrose do miocárdio em muitas cardiomiopatias, incluindo na doença de Chagas. Estudos iniciais incluindo pacientes com cardiomiopatia chagásica em diferentes estágios da doença claramente demonstraram que quanto maior a gravidade da doença maior a quantidade de fibrose do miocárdio. Com base nesses dados e na história natural da doença, caracterizada pela progressão da disfunção ventricular esquerda, pode-se inferir, seguramente, que a fibrose miocárdica evolui com o tempo em todos os pacientes. Contudo, a obtenção de dados longitudinais utilizando RMC em pacientes com doença de Chagas não era disponível até a publicação do artigo original de Santos et el.,^5^ nesta edição dos Arquivos Brasileiros de Cardiologia. Esse é o primeiro estudo incluindo dados sobre fibrose miocárdica obtidos de dois exames consecutivos de RMC do mesmo paciente, com um período relativamente longo de acompanhamento, de 5,4 anos. Os autores demonstraram um aumento expressivo de 43% na fibrose miocárdica durante o período de acompanhamento, o que indica um acréscimo médio de 7,9% ao ano de fibrose do miocárdio detectado por RMC.

Apesar da originalidade do resultado, o qual, possivelmente foi um gerador de hipótese, esse foi um estudo retrospectivo incluindo uma amostra pequena de 20 pacientes. O estudo também investigou a associação entre fibrose do miocárdio e função ventricular esquerda, o que já foi demonstrado em estudos prévios. Isso, no meu ponto de vista, tirou o foco da evolução da fibrose do miocárdio, o que era, na realidade, a informação original do artigo, e que merecia ser mais bem explorado. Por isso, outros estudos maiores são mandatórios para expandir o conhecimento acerca desse parâmetro crucial que contribuiria para o entendimento da fisiopatologia da doença e o desenvolvimento de novos tratamentos da cardiomiopatia chagásica.

Os mecanismos evolvidos na evolução da fibrose do miocárdio, incluindo aspectos da microcirculação do miocárdio^6^ e das artérias coronárias epicárdicas,^7^ e mesmo envolvimento de vias metabólicas específicas, não foram discutidos no artigo.^8^

Um resultado surpreendente do estudo foi a falta de associação de fibrose com idade e sexo, a qual foi demonstrada em um estudo prévio.^9^ Tal fato, mais uma vez, deve-se ao tamanho amostral muito pequeno.

Outro aspecto interessante da cardiomiopatia chagásica é a baixa frequência de fibrose do miocárdio observada na fase aguda da doença de Chagas (comunicação pessoal de João Marcos Ferreira) e o possível surgimento de novas anomalias cardíacas ao longo do seguimento.^10^ Isso pode indicar que a fibrose miocárdica na doença de Chagas seja um processo implacável, dependendo do tempo e da intensidade do processo inflamatório. Nesse sentido, um estudo prévio também mostrou que, diferentemente da fibrose isquêmica, a fibrose causada por cardiomiopatia chagásica está associada com edema do miocárdio detectado por imagens ponderadas em T2 na RMC, indicando presença de inflamação.

A fibrose miocárdica na doença de Chagas não é um mero processo espectador passivo, e sim, um processo dinâmico, revelador, que leva à progressiva lesão do miocárdio e inflamação. Esse conceito é essencial para o desenvolvimento de métodos que interrompam essa via letal.

Embora o estudo publicado nesta edição dos Arquivos Brasileiros de Cardiologia^5^ não tenha sido delineado para investigar o mecanismo de progressão da fibrose miocárdica, esse conhecimento é crucial se desejamos expandir nosso conhecimento acerca da fisiopatologia e tratamento da cardiomiopatia chagásica em um futuro próximo.
