# Supplementary material for: A Importância de se Entender a Evolução da Fibrose Miocárdica na Cardiomiopatia Chagásica Crônica
Source: Arq Bras Cardiol. 2021 Nov 22;117(6):1091–2. [Article in Portuguese] doi: 10.36660/abc.20210897 (PMC8757144; doi:10.36660/abc.20210897)
Supplement: Translation

# Translation: The Importance of Understanding the Progression of Myocardial Fibrosis in Chronic Chagas Cardiomyopathy

**Affiliations:** 1 São Paulo SP Brazil Universidade de São Paulo, Faculdade de Medicina, Hospital das Clínicas - Instituto do Coração, São Paulo, SP - Brazil; 2 São Paulo SP Brazil Hospital do Coração (HCOR), São Paulo, SP - Brazil

**Keywords:** Chagasic Cardiomyopathy, Endomyocardial Fibrosis, Chagas Diseases, Heart Failure, Diagnostic, Imaging/méthods, Imaging Resonance Magnetic/methods

Myocardial fibrosis is one of the most important biological markers of Chagas’ cardiomyopathy and is directly related to the severity of the cardiomyopathy and the stage of the disease.^1^ In addition, myocardial fibrosis has prognostic value for major cardiovascular events, with a strong and close relationship with arrhythmic events in these patients.^2-4^

Cardiac magnetic resonance (CMR) is the reference imaging method for myocardial fibrosis detection and quantification in many cardiomyopathies, including Chagas cardiomyopathy. Initial studies included patients with Chagas cardiomyopathy at different stages of the disease and clearly demonstrated that the higher the disease severity the larger the amount of myocardial fibrosis. Based on these data and on the natural history of the disease, with progression of left ventricle dysfunction, one could safely infer that myocardial fibrosis progresses with time for any given patient. However, the longitudinal data using CMR in patients with Chagas disease was not available until the publication of the original article in this issue of ABC Cardiol by Santos et al.^5^ This is the first study to include data on myocardial fibrosis from two consecutive CMR of the same patient with a relatively long follow-up of 5.4 years. The authors demonstrated an impressive 43% increase of myocardial fibrosis over the follow-up period, which indicates a mean of 7.9% increase per year of myocardial fibrosis detected by CMR.

Despite the originality of this finding, which was perhaps, a hypothesis generator, this is a retrospective study with a rather small sample only 20 patients. The study also investigated the association of myocardial fibrosis with left ventricle function, which has already been demonstrated in previous studies. This, in my view, took the focus away from myocardial fibrosis progression, which was the original information of the manuscript and should have been explored more deeply and in more detail. I understand the limited sample size might not allow such a detailed analysis. For this reason, additional and larger studies are mandatory to further the knowledge on this crucial parameter that could be the “the one” to help us to understand the pathophysiology and develop new treatments for Chagas cardiomyopathy.

Mechanisms involved in the progression of myocardial fibrosis, including aspects of the myocardial microcirculation,^6^ status of the epicardial coronary arteries^7^ and even involvement of specific metabolic pathways^8^ were not discussed in this manuscript.

A surprising result of the study was the lack of association of fibrosis with age and gender, which has been demonstrated in previous work.^9^ Again, this might be the result of very small sample size.

Another interesting aspect of Chagas cardiomyopathy is the low frequency of myocardial fibrosis observed in the acute phase of Chagas disease (personal communication by João Marcos Ferreira) and the possible appearance of new cardiac abnormalities over the follow-up.^10^ This may indicate that myocardial fibrosis in Chagas disease is a relentless process, depending on time and the intensity of the inflammatory process. In this regard, prior work has also shown that, differently from ischemic fibrosis, Chagas cardiomyopathy fibrosis is associated to myocardial edema detected by T2- weighted CMR images, indicating the presence of inflammation.

Myocardial fibrosis in Chagas disease is not a fixed and bystander process, but rather, a dynamic and unfolding process leading to progressive myocardial injury and inflammation. This concept is essential for developing methods to try to stop this lethal pathway.

Although the study published in this issue of ABC Cardiol^5^ was not designed to investigate the mechanism of the progression of myocardial fibrosis, this knowledge is crucial if we want to advance our knowledge in the pathophysiology and treatment of Chagas cardiomyopathy in the early future.
